# Global Perspective on the Development of Genetically Modified Immune Cells for Cancer Therapy

**DOI:** 10.3389/fimmu.2020.608485

**Published:** 2021-02-15

**Authors:** Laetitia Pinte, Amy Cunningham, Helene Trébéden-Negre, Sarah Nikiforow, Jerome Ritz

**Affiliations:** Connell and O’Reilly Families Cell Manipulation Core Facility, Dana-Farber Cancer Institute, Harvard Medical School, Boston, MA, United States

**Keywords:** chimeric antigen receptor T cells, T cell receptor, cell therapy, genetically modified cells, clinical trials

## Abstract

Since the first genetically-engineered clinical trial was posted to *clinicaltrials.gov* in 2003 (NCT00019136), chimeric antigen receptor (CAR) and T-cell receptor (TCR) therapies have exhibited unprecedented growth. USA, China, and Europe have emerged as major sites of investigation as many new biotechnology and established pharmaceutical companies invest in this rapidly evolving field. Although initial studies focused primarily on CD19 as a target antigen, many novel targets are now being evaluated. Next-generation genetic constructs, starting materials, and manufacturing strategies are also being applied to enhance efficacy and safety and to treat solid tumors as well as hematologic malignancies. Fueled by dramatic clinical efficacy and recent regulatory approvals of CD19-targeted CAR cell therapies, the field of engineered cell therapeutics continues to expand. Here, we review all 745 genetically modified CAR and TCR clinical trials with anticipated accrual of over 28,000 patients posted to *clinicaltrials.gov* until 31^st^ of December 2019. We analyze projected patient enrollment, geographic distribution and phase of studies, target antigens and diseases, current strategies for optimizing efficacy and safety, and trials expected to yield important clinical data in the coming 6–12 months.

## Introduction

Genetic engineering of immune cells to express defined antibody-based chimeric antigen receptors (CAR) has led to highly effective cell-based therapeutics for cancer. Several groundbreaking preclinical and clinical efforts in the late 1980’s and 1990’s laid the groundwork for modern CAR and TCR clinical investigation. These include clinical studies of ex vivo expanded tumor infiltrating lymphocytes (1) and the first chimeric receptors developed by Kurosawa, Eshhar, and colleagues (2–4). When combined with advances in viral transduction of immune cells (5–7) and the incorporation of costimulatory domains with receptor constructs (8–10) these different streams of scientific discovery enabled the dramatic number of CAR and T cell receptor-based (TCR) therapies that are currently ongoing. This foundational work has been previously well-reviewed (11–13). Now, already in clinic for over a decade, anti-CD19 CAR-T cells have shown remission rates as high as 90% in pediatric ALL and 50%–90% in adult B-cell malignancies (14–16). Remarkable efficacy in patients with multiply relapsed or refractory disease led to approval of tisagenlecleucel and axicabtagene ciloleucel anti-CD19 CAR-T therapies for relapsed B cell leukemia and lymphoma, in the United States, Europe and Japan (17–20). In July 2020, the US FDA granted accelerated approval to brexucabtagene autoleucel for relapsed adult mantle cell lymphoma (21). Additional anti-CD19 CAR-T constructs are in late stage development, and Phase III trials are now comparing CAR products with standard second-line therapies. The results of these studies may further extend the indications for anti-CD19 CAR-T therapy to patients prior to developing highly resistant tumor cells and complications from prior therapy.

The success of CAR-T therapies has sparked a worldwide surge of clinical trials seeking to improve safety and efficacy and identify new disease indications for genetically-engineered immune cell therapies. To quantitatively explore the evolution of this new treatment modality, we generated a database encompassing all CAR and TCR-based interventional clinical trials posted on *clinicaltrials.gov* from the first trial recorded in 2003 to the last one registered in 2019.

## Methods

Clinical trials involving TCR and CAR therapies were extracted from *clinicaltrials.gov*. Data were then sorted and analyzed manually for quality control purposes. Only interventional trials posted prior to December 31, 2019 were selected. Trials investigating lymphocyte-based therapies not featuring genetic modifications with CARs or TCRs, such as antigen-expanded cytotoxic T lymphocytes or tumor-infiltrating lymphocytes, were excluded from this report. Average patient numbers were rounded to the closest and smaller integer. Of note, reported patient enrollment indicates anticipated enrollment over the full course of each trial and does not reflect actual numbers of patients enrolled at any given time point. Country totals reflect the location of anticipated patient enrollment to the clinical trial and not the sponsor’s country of origin. Clinical trial phases and sponsors were defined as described in **Supplementary Tables 1**and**2**. Trial status, e.g. active, recruiting, not yet recruiting, was not collected in this analysis. *Clinicaltrials.gov* data is limited by what end-users disclose in trial entries and not all trials are registered. If a parameter was omitted in a *clinicaltrials.gov* entry, the trial was excluded from reported totals for that variable, e.g. a CAR study with unspecified target was excluded from tables summarizing number of trials per target. Additional information regarding these trials may be publicly available but was not included in this analysis. Other databases such as EudraCT also catalogue clinical trials conducted with genetically modified immune cells. Since these databases largely overlap with *clinicaltrials.gov*, we limited our analysis to trials listed in *clinicaltrials.gov*.

## Seventeen-Year History of Clinical Investigation

**Figure 1:**
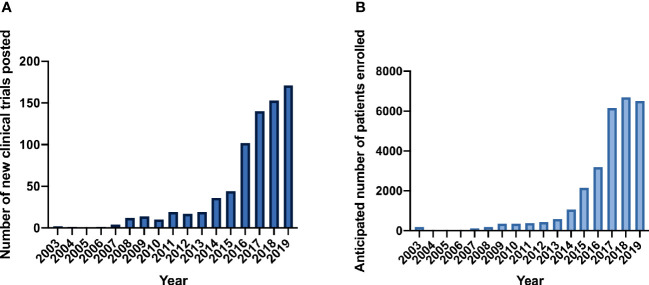
CAR and TCR clinical trials on *clinicaltrials.gov*. **(A)** New clinical trials posted per year. **(B)** Anticipated patient enrollment per year.

Since the first genetically-engineered T-cell trial was listed on *clinicaltrials.gov* in 2003, 745 total CAR and TCR clinical trials have been registered (**Figure 1A**). Between 2003 and 2007, only eight trials were listed. Studies increased in the next 5-year period 2008-2012, in which 72 new clinical trials were posted. Annual new trials rose dramatically beginning in 2014 (**Figure 1A**). In the most recent 5-year period, 608 new trials were registered on *clinicaltrials.gov*. Reported patient enrollment reflected trends observed in the number of new clinical trials (**Figure 1B**). (Of note, reported patient enrollment on *clinicaltrials.gov* may not reflect actual patient totals on trial). In the early period from 2003 to 2008, only 306 patients were anticipated to enroll in a limited number of clinical trials. From 2009 to 2014, expected eventual enrollment across all trials increased to 1,709 patients. This number rose dramatically in the last 5-year period in which 24,695 patients were anticipated in CAR and TCR trials.

**Figure 2:**
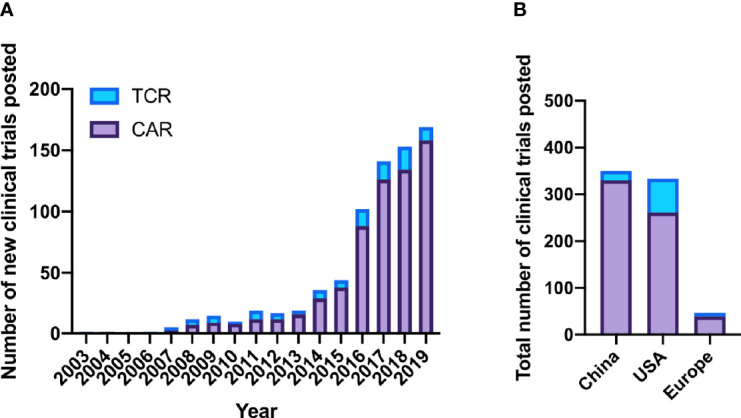
CAR vs. TCR clinical trials posted on *clinicaltrials.gov*. **(A)** CAR and TCR clinical trials posted per year. **(B)** Total CAR and TCR trials by location 2003-2019.

Overall, 641 CAR clinical trials were listed compared to 102 TCR-based therapies, while two trials involved dual CAR and TCR therapies. Although genetically-engineered TCR trials predominated from 2003 to 2008, the sharp increase in clinical investigation that began in 2014 focused on CAR trials (**Figure 2A**). This trend continued in 2019, when 158 new CAR trials were listed while only 11 new TCR trials were started.

## Evolving International Landscape of Genetically Modified Cell Therapies

To better understand where CAR and TCR trials have taken place, we analyzed clinical trial postings by geographic area. Forty-seven percent of overall trials were based in China, 44% from USA, and 6% European (**Figure 2B**). Other countries including Japan, Australia, Canada, Malaysia, New Zealand, and Israel hosted few trials (n=14, collectively). While most CAR trials opened in China (n=330 of 641), overtaking USA in 2015, most TCR studies were conducted in the USA (n=69 of 102).

**Figure 3:**
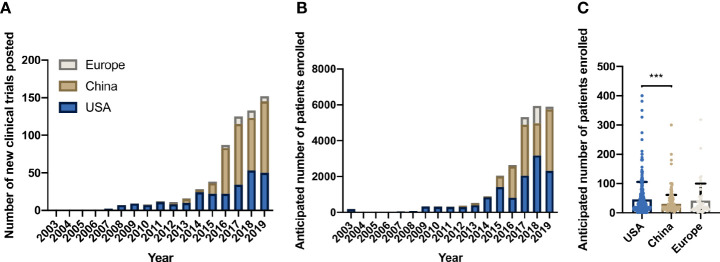
Location of CAR trials on *clinicaltrials.gov*. **(A)** New CAR trials per year by location. **(B)** Anticipated patient enrollment in CAR trials per year by location. **(C)** Comparison of total anticipated patient enrollment by location 2003-2019 (p=0.0003, one-way ANOVA test). *** denotes p-value < 10^-3^.

From 2003 to 2007 only eight trials were listed in *clinicaltrials.gov*, four CAR trials and four TCR-based studies, all conducted in the USA. The first CAR trial began in the USA in 2003. Registered CAR studies opened in Europe in 2010, and China entered the field in 2012 (**Figure 3A**). From 2012 to 2015, China hosted 23 CAR trials. In 2016 alone, 61 CAR trials occurring in China were registered. Since 2016, more CAR trials have opened in China than in USA (307 vs. 159, respectively). During this same period, 31 new CAR trials began in Europe. Following this trend, trials in China reported the highest annual patient enrollment since 2016, followed by USA and Europe (**Figure 3B**). Though most CAR clinical trials took place in China, average expected patient enrollment per trial was significantly lower than in USA trials (p-value = 0.0003, **Figure 3C**). This difference was especially striking for late phase trials (phase II/III and III). USA opened five late phase trials with average anticipated enrollment of 208 patients, while China had five late phase trials, averaging 18 reported patients per trial.

From 2003 until 2013 academic sponsors supported the vast majority of trials (91%). Subsequently, industry became more engaged in the field and commercial entities are now the predominant sponsors of both CAR and TCR-based clinical trials. In the last 5 years, 54% of studies had industry sponsors and 46% had academic sponsors. This trend is particularly evident in China, where 55 different companies funded CAR and TCR trials versus 34 in USA and 12 in Europe from 2003 to 2019. This may reflect differences in regulatory requirements between countries and Chinese initiatives such as “Made in China 2025” targeting investments in research and development (22, 23). Across all countries, industry sponsored nearly half of all CAR (n=301) and TCR trials (n=42) (**Supplementary Table 2**). International collaborations and mergers have supported the worldwide increase in genetically-modified immune therapies, largely in CAR products. In 2017, Johnson & Johnson invested $350 million in the Chinese Biotech Nanjing Legend Biotechnology Co. for the global rights to co-develop and market experimental CAR treatments (24) and Novartis invested $40 million into Cellular Biomedicine Group in USA (25). Major corporate buyouts of USA CAR manufacturers also occurred. Gilead bought Kite Pharma in August 2017 (26), and Celgene bought Juno Therapeutics in January 2018 (27). Bristol Myers Squibb then acquired Celgene in 2019 (28).

## Most Studies Continue to Be Early-Phase Clinical Trials

CAR trials posted from 2003 to 2015 were all early-phase studies (phase I, I/II, or II), which focused on patients with relapsed/refractory disease with few treatment options and did not include comparator cohorts. While the dramatic efficacy of anti-CD19 CAR-T cells demonstrated in phase II trials was sufficient for regulatory approval, randomized trials were initiated in 2018 to extend their clinical indications. Late phase studies were distributed across USA, China, and Europe (n=5, 5, and 3, respectively). USA and Europe held the highest total anticipated patient enrollment in late phase trials (n=1041, 558 respectively vs. 90 in China). The first randomized phase III trial opened in January 2018, a study of Kite’s axicabtagene ciloleucel anti-CD19 CAR-T cells in adult relapsed/refractory diffuse large B cell lymphoma vs. standard of care second-line salvage chemotherapy plus autologous stem cell transplant (ASCT) (NCT03391466, **Table 1**). In 2018, Novartis initiated phase III randomized trials of tisagenlecleucel anti-CD19 CAR-T versus standard of care second-line therapies (such as blinatumomab or inotuzumab ozogamicin) in adult patients with relapsed/refractory hematological malignancies (NCT03628053, NCT03570892). Celgene also launched phase III trials the same year to test its anti-CD19 CAR-T in Non-Hodgkin’s Lymphoma (NCT03575351) and BCMA-CAR T in multiple myeloma, (NCT03651128) in comparison to standard therapies (salvage chemotherapy and ASCT or later-generation Imids/proteasome inhibitors and/or daratumumab, respectively). In 2019, Janssen initiated a phase III study of its anti-BCMA CAR-T vs. combination pomalidomide, bortezomib, dexamethasone or daratumumab, pomalidomide and dexamethasone in lenalidomide-refractory adult myeloma (NCT04181827). In July 2019, the first late phase trial involving CAR-T cells in solid tumors was listed, a phase IIb study of an anti-CEA CAR combined with either nanoparticle albumin-bound paclitaxel, 5-fluorouracil/folinic acid, or capecitabine vs. chemotherapy alone in CEA+ pancreatic adenocarcinoma liver metastases (NCT04037241). In contrast, all TCR trials continue to be early-phase, and none have demonstrated sufficient efficacy to garner fast-track approval to date. That the clear majority of clinical trials continue to be early phase studies indicates the field of genetically-engineered cellular therapeutics remains relatively young, with great emphasis on innovation and development of new cellular products. These products will require demonstration of safety and preliminary efficacy before they can expand to large multicenter trials that include comparisons with standard treatments.

**Table 1:** CAR therapies compared to standard of care.

NCT#	Year posted	Phase	CAR-target	Company	Disease	Anticipated Patient Enrollment
NCT03391466	2018	3	CD19	Gilead/Kite	DLBCL	350
NCT03628053	2018	3	CD19	Novartis	ALL	220
NCT03570892	2018	3	CD19	Novartis	NHL	318
NCT03575351	2018	3	CD19	Celgene	NHL	182
NCT03651128	2018	3	BCMA	Celgene	MM	381
NCT04181827	2019	3	BCMA	Janssen	MM	95

Long-term clinical data is emerging for early anti-CD19 CAR-T constructs. Two-year follow-up of patients receiving axicabtagene ciloleucel in Zuma-1 (NCT02348216) indicates the drug can induce durable responses and median overall survival greater than 2 years with manageable long-term safety in adult large B-cell lymphoma (29). Follow-up studies of patients receiving commercial axicabtagene ciloleucel and tisagenlecleucel confirm the remarkable efficacy and manageable safety profiles of these drugs beyond the clinical trial setting (30–33). Though not yet commercially approved at the time of this review, long-term trial data from 269 adult B-cell lymphoma patients receiving lisocabtagene maraleucel, an anti-CD19 CAR-T cell therapy, indicated 18.8 month median overall survival, 73% objective response rate, 53% complete response rate, and 12% occurrence of grade 3 or worse cytokine release syndrome or neurological events (NCT02631044) (34). However, across studies it appears there is still room for optimization of these therapies. Late phase randomized studies may help to refine treatment algorithms, perhaps allowing for patients to receive CAR therapies as earlier treatment options and further identifying prognostic indicators to help precisely identify patients who would most benefit from particular CAR therapies.

## CAR and TCR Targets

The first successful CAR-T cells targeted CD19 expressed on pre-B ALL, B-cell lymphomas, and normal B cells. Two-hundred fifty-five CAR clinical trials enrolling an anticipated 11,783 patients targeted CD19 (**Table 2A**). The next most frequent target in hematologic malignancies was BCMA in multiple myeloma, with 2,517 patients expected to enroll in 47 single-agent BCMA CAR trials. The clinical efficacy of BCMA CAR-T cells has been noted in several studies in relapsed/refractory myeloma, and FDA approval is expected for several such products in the near future (35–38). CD22 has emerged as a new CAR target in B-ALL and B-cell lymphoma (n=15) and CD30 in relapsed Hodgkin’s disease and T-cell lymphomas (n=15). Overall, CARs targeting 23 different surface antigens expressed by hematologic malignancies have been evaluated in clinical trials (**Table 2A**). Many of these antigens are expressed selectively by normal B cells and B-cell malignancies (ALL, CLL, B-cell lymphoma, myeloma). In these cases, successful therapy also results in the elimination of normal B cells or plasma cells, and patients are supported by administration of normal human gamma globulin until normal B cells recover sufficiently to produce endogenous antibodies. Several recent trials also target antigens expressed on T-cell or myeloid malignancies. In these cases, CARs may also target normal T cells or normal hematopoietic stem cells, potentially resulting in severe immune deficiency or life-threatening pancytopenia. In many of these trials, treatment is planned as a bridge to allogeneic stem cell transplantation. In other cases, innovative approaches are designed to mitigate these toxicities.

**Table 2A:** Single targets for CAR therapies in liquid tumor indications.

Target	Number of clinical trials	Expected patient enrollment
**CD19**	255	11783
**BCMA**	47	2517
**CD22**	15	472
**CD30**	15	391
**CD123**	14	535
**CD20**	11	378
**CD33**	6	136
**CS1**	3	102
**CD7**	4	155
**NKG2DL**	4	194
**CD138**	2	43
**CD4**	2	72
**LeY**	2	51
**CD133**	1	20
**CD37**	1	34
**CD38**	1	72
**CD44v6**	1	10
**CD5**	1	21
**FLT3**	1	35
**KLC of Ig**	1	54
**PD-L1**	1	20
**ROR**	1	0
**TRBC1**	1	55

Although most single-target CARs were specific to antigens expressed primarily on hematopoietic malignancies (n=390), many solid tumor CARs were also evaluated (n=156, **Table 2B**). The first solid tumor CAR trial was posted on *clinicaltrials.gov* in 2003 and utilized anti-L1-CAM (CD171) CAR CD8+T cells to treat patients with neuroblastoma (NCT00006480) (39). Other early solid tumor trials include an anti-GD2 CAR-T trial in neuroblastoma in 2004 (NCT00085930) (40) and an anti-CAIX CAR-T in renal cell carcinoma not registered on *clinicaltrials.gov* but published in 2006 (41). For all trials listed on *clinicaltrials.gov*, 32 different solid-tumor antigens have been evaluated as targets in early-phase clinical trials. Mesothelin has been the most frequently targeted antigen in solid-tumor CAR trials (n=22), followed by GD2 (n=17), and EGFR (n=16). Current and prospective solid-tumor targets have been previously well reviewed (42, 43). Identification of appropriate target antigens is particularly important in solid tumors. Target antigens expressed at high density on solid tumors are often also expressed on normal tissues and are likely to result in unacceptable toxicities when attacked by CAR-T cells *in vivo* (42, 43). One approach to address this limitation focuses on tumor-specific splice variants of proteins such as EGFRvIII, which has been evaluated in 8 different trials (44). Although CARs directed at many solid-tumor antigens have been evaluated and clinical responses have been observed, the clinical efficacy of these products has thus far been modest. Overall results in these trials are not comparable to the remission rates achieved in patients with B-cell malignancies (45–47).

**Table 2B:** Single targets for CAR therapies in solid tumor indications.

Target	Number of clinical trials	Expected patient enrollment
**Mesothelin**	22	654
**GD2**	17	528
**EGFR**	16	379
**HER2**	14	463
**GPC3**	13	330
**CEA**	12	260
**MUC1**	9	399
**PSMA**	7	140
**EpCAM**	5	174
**IL13Rα2**	5	201
**NKG2DL**	5	144
**CLD18**	3	60
**ROBO1**	3	42
**B7-H3**	2	80
**c-Met**	2	16
**CD147**	2	54
**PD-L1**	2	42
**PSCA**	2	150
**ROR**	2	148
**AFP**	1	18
**CD171**	1	40
**CD20**	1	18
**CD70**	1	113
**Chlorotoxin**	1	18
**EphA2**	1	60
**FAP**	1	4
**Folate receptor**	1	18
**GP100**	1	6
**LeY**	1	82
**LMP**	1	20
**MUC16**	1	30
**VEGFR2**	1	24

In contrast to CARs specific for cell surface molecules, TCR-based therapies are directed against peptide epitopes presented in the context of the patient’s individual HLA molecules. This approach has the advantage of targeting intracellular proteins, including unique tumor neoantigens and tumor-associated antigens that are not widely expressed on normal tissues. However, TCR specificity is HLA-restricted, and trial enrollment is therefore limited to patients (and tumors) that express specific HLA alleles. NY-ESO-1 has been the most frequent antigen targeted in TCR trials (n=34), followed by MAGE (n=9, **Table 3A**) (14). Eighty percent of TCR trials (79 of 102) are restricted to patients who are HLA-A02 positive. HLA-A11 and HLA-A24 were the next most prevalent eligible HLA alleles, included in 8 and 5 trials, respectively. Only 10 TCR studies targeted hematopoietic tumor antigens, mainly WT1 (n=3) and NY-ES0-1 (n=3). In December 2019, results of a phase I trial featuring a TCR specific to peptides shared by NY-ESO-1 and LAGE-1 yielded a 42% overall response rate 1-year post infusion in multiple myeloma (NCT01352286) (48). In efforts to reduce on-target off-tumor toxicities, TCRs have also been designed for cancer-specific mutations, like TGF-β receptor II polymorphisms in colorectal cancer (NCT03431311), EBV-LMP1 epitopes that are highly expressed in EBV-associated nasopharyngeal carcinoma (NCT03648697), and KRAS G12V in pancreatic cancer harboring that mutation (NCT04146298).

**Table 3A:** Single targets for TCR therapies.

Target	Number of clinical trials	Expected patient enrollment
**NY-ESO-1**	34	526
**MAGE**	9	313
**HPV Ag**	6	556
**MART-1**	4	100
**WT1**	4	65
**HBV Ag**	3	38
**KRAS**	3	191
**CEA**	2	17
**CMV Ag**	2	19
**EBV Ag**	2	45
**gp100**	2	24
**HIV Ag**	2	26
**P53**	2	15
**AFP**	1	9
**Folate Receptor**	1	0
**HER2**	1	33
**HERV**	1	24
**MCP Ag**	1	16
**Mesothelin**	1	10
**TGFbRII**	1	5
**TRAILxDR4**	1	5

Though anti-CD19 and anti-BCMA CARs frequently induced disease remission, some patients relapse after initial response. The most frequent reasons for tumor relapse have been either lack of persistence of genetically-modified effector cells *in vivo* or loss of epitope expression by the tumor cells (49). In a phase IIa single agent anti-CD19-CAR trial in adult NHL, post-infusion biopsies showed CD19 antigen loss in 20% of non-responding patients (NCT02030834) (15). Similarly, CD19 epitope loss occurred in 28% of pediatric patients who received anti-CD19 CAR-T cells (14, 50). This demonstration has led to a series of new clinical trials using CARs targeting 2 different surface antigens as a strategy to prevent relapse due to antigen loss.

Eighteen different dual CAR constructs that target multiple antigens simultaneously have now entered clinical trials (**Table 2C**). Multiantigen-specific CARs may have three or more targets but typically have two. Anti-CD19/CD22 (n=17) and anti-CD19/CD20 (n=13) combinations were most frequently tested. Several multiantigen-specific TCRs have also been developed, mainly NY-ESO-1/LAGE-1a (n=4) (**Table 3B**). In a different approach, two trials explored a modulable receptor using a CD16v CAR co-administered with monoclonal antibodies (mAb). By enabling generic antibody binding through CD16, this strategy allowed targeting of different diseases by interchanging commercially available mAbs like anti-CD20 (NCT02776813) and anti-HER2 (NCT03680560).

**Table 2C:** CAR therapies targeting multiple antigens.

Target	Number of clinical trials	Expected patient enrollment
**CD19/CD22**	17	598
**CD19/CD20**	13	275
**CD19/BCMA**	5	85
**BCMA/CS1**	1	84
**BCMA/CD138**	1	10
**BCMA/CD38**	1	80
**CD20/CD22/CD10**	1	30
**CD19/CD20/CD22/CD30**	1	10
**CD20/CD3**	1	12
**CD123/CLL1**	1	20
**CD123/CD33**	1	10
**CD33/CLL1**	1	70
**c-MET/PDL1**	1	50
**Mesothelin/CD19**	1	4
**Muc1/CLL1/CD33/CD38/CD56/CD123**	1	10
**Nectin4/FAP**	1	50
**PD-L1/CD80/CD86**	1	10

**Table 3B:** Multiple targets for TCR therapies.

Target	Number of clinical trials	Expected patient enrollment
**NY-ESO-1/LAGE-1a**	4	132
**AFP/HLA-A2**	1	24
**HER2/CD3**	1	8
**gp100/CD3**	1	327
**gp100/MART-1**	1	4
**Melan-A/MART1**	1	12

## Boosting Cell Survival and Efficacy Through Additional Genetic Modifications

Prolonged disease remission and survival are associated with immune effector cell persistence in the tumor microenvironment (51). Immune checkpoint signaling, hypoxia, and metabolic milieu of the tumor microenvironment all promote immune evasion and tumor survival (52). As summarized in **Table 4** and **Supplementary Table 3**, numerous strategies in the last 4 years have been used to enhance functionality of CARs and TCRs in the tumor microenvironment, including: (1) resistance to exhaustion/negative regulation, (2) secretion of cytokines, and (3) enhanced tumor homing.

**Table 4:** Novel genetic modifications in CAR and TCR therapies from 2016 to 2019.

	CAR		TCR		
***RESISTANCE TO NEGATIVE REGULATION***	PD-1 knock-out	n = 6	PD-1 knock-out	n = 1	
	Anti-PD-1 expression	n = 5	Anti-PD-1 expression	n = 2	
	PD-L1 blocker expression	n = 5			
	Anti-PD-1 and anti-CTLA-4 expression	n = 3			
	PD-1 Fc fusion protein secreted	n = 2			
	PD-1 shRNA expression	n = 1			
	Activated cytoplasmic PD-1	n = 1			
	TGF-beta resistance	n = 1			
	Endogenous HPK1 disruption	n = 1			
***INTERLEUKIN EXPRESSION***	IL15	n = 5			
	IL-7 and CCL19	n = 2			
	IL-7 receptor	n = 2			
	IL-7 and CCL19 or IL12	n = 1			
	IL-15 or both IL-15 and IL-21	n = 1			
	IL-12	n = 1			
***TUMOR HOMING***	CCR4 expression	n = 1			
	CXCR5 modified	n = 1			
***FRATRICIDE RESISTANCE***	CD7 knock-out CD7-targeting CAR	n = 1			
***SAFETY SWITCH***	iCasp9	n = 13	iCasp9	n = 1	
	EGFRt	n = 13			
	EGFRt and HERt on two different CARs	n = 1			
	Herpes simplex virus thymidine kinase	n = 1			
	RQR8	n = 1			
***VIRUS RESISTANCE***	Resistant to HIV by CCR5 modification	n = 1			
***IMMUNE RESISTANCE***	β2m and TCR disruption	n = 1	TCRα β disruption		n = 1

The most frequent approaches entailed PD-1 knockout (n=6 CAR, n=1 TCR) and genetic modification to effect local secretion of anti-PD-1 (n=5 CAR, n=2 TCR). Five trials describing CARs with PD-L1 blockers featured constructs that rewired PD-1 extracellular domains to an internal activating domain (n=5) (53). Three trials studied CARs that could locally secrete anti-PD-1 and anti-CTLA-4 antibodies (n=3). Other modifications included a PD-1 Fc-receptor-like fusion protein (n=2), shRNA suppression of PD-1 expression (n=1), activated cytoplasmic PD-1 (n=1), conferring resistance to TGF-β (n=1), and CRISPR-mediated knockout of *HPK1* in anti-CD19 CAR-T (NCT04037566) (54, 55).

Inducing local cytokine secretion was another approach found in CAR trials (n=12) to modify the tumor microenvironment and enhance effector cell activity *in vivo*. Local cytokine secretion by activated effector cells further avoids potential toxicities of systemic cytokine infusion. More than half of these trials targeted solid tumors (n=7), but no TCR trials employed this strategy. Five trials leveraged IL-15 secretion to promote expansion and survival of memory T cells and enhance NK-cell mediated cytotoxicity, and one trial used both IL-15 and IL-21 (56, 57). Two trials explored the expression of IL7/CCL19 alone, while two others tested direct expression of IL-7 receptor to enhance cell survival. IL-12 secretion by CAR cells was used for local dendritic cell maturation and T-cell proliferation with or without IL-7/CCL19 (NCT03542799, NCT03932565). To enhance trafficking to tumors, CARs have been engineered to express chemokine receptors in two trials: CCR4 in anti-CD30 CAR-T for cutaneous lymphoma (NCT03602157) and CXCR5 co-expressed with anti-EGFR CAR-T in non-small cell lung cancer (NCT04153799).

The ability to use CAR-T cells to target T-cell tumors has been complicated by the fact that normal T cells also express the same target antigens. This results in fratricide and loss of CAR-T cells during manufacturing and *in vivo* after infusion (58). To avoid this cross-reactivity, one trial (NCT03690011) used CRISPR-based gene editing to knockout CD7 in T cells prior to CD7-CAR transduction of CAR-T for T-cell malignancies. While that trial was the only one to employ fratricide resistance modifications, recent results of a phase I CD5 CAR-T trial in r/r T-cell leukemia and lymphoma showed no sign of T-cell fratricide (NCT03081910) (59).

## Increasing Safety of Genetically-Engineered Cells

Along with remarkable efficacy, CAR-T cell therapies have also been associated with serious toxicities directly related to rapid expansion and activation of large numbers of activated effector cells *in vivo*. This has led to the development of different approaches to rapidly and selectively eliminate CAR-T cells *in vivo* in patients with life-threatening toxicities. One approach relies on genetic modifications such as “safety switches” that induce expression of drug-targetable molecules leading to rapid elimination of modified cells. Twenty-nine trials featured this strategy; 28 were CAR therapies, of which 22 were conducted in the USA. Safety switch triggers were based predominately on already approved drugs with known, manageable adverse effects and were, to date, studied in CARs and one TCR trial (**Table 4** and **Supplementary Table 3**). The most studied method required rimiducid, which prompts dimerization of caspase-9 (iCasp9) to induce apoptosis (n=13 CAR, 1 TCR). Remaining strategies were studied only in CAR trials. Another safety switch mechanism relied on forced expression of non-functional surface molecules that can be targeted by systemic administration of monoclonal antibodies, like truncated endothelial growth factor receptor (EGFRt) (n=13, 2 allogeneic trials), a target of cetuximab-driven antibody-dependent cell-mediated cytotoxicity (60). One trial studied EGFRt and the related truncated human epidermal growth factor receptor 2 (HERt) on two different CAR constructs. Cells edited to express herpes simplex virus-1 thymidine kinase (HSV-TK) were found in one allogeneic CAR trial and could be systemically killed upon ganciclovir administration. A construct called RQR8, which combines CD34 and CD20 target epitopes, enabling both a CD34 selection to enrich edited cell populations in manufacturing and a rituximab-induced safety-switch, was found in one trial (NCT03590574) (61). Select allogeneic CAR trials are also known to employ a rituximab-targetable CD20 mimic embedded in CAR constructs, though the trial entries did not specify use of this kill switch molecule on *clinicaltrials.gov* (NCT03203369, NCT03190278, NCT04142619, NCT04150497, NCT04106076, NCT03229876, NCT02746952) (62).

## Alternate Cells of Origin

Prior to 2019, cells used for CAR and TCR manufacturing were typically T cells sourced from autologous leukapheresis products (96% of trials). Rarely, cord blood was used as a source of allogeneic immune cells (2 CAR-NK and 1 CAR-T cell trials). The T-cell subsets infused affects CAR-T efficiency and persistence (63). Very few trial listings specify detailed information on the composition of the CAR T cell products. Central memory cells are implicated in longer persistence and survival of adoptively-transferred immune cells and were selectively utilized in at least 4 CAR trials (64). γδT cells, which can target cells in an MHC-independent manner and have been implicated in anti-tumor responses with less cytokine release and functional exhaustion than in TCRαβ signaling, have been selectively utilized in allogeneic CAR trials (n=2) (47, 65, 66). Natural Killer (NK) cell CARs have also been investigated (n=21 since 2016). NK cells express multiple innate MHC-independent activating receptors (e.g. NKG2D, natural cytotoxicity receptors, and DNAM-1) that respond to commonly upregulated ligands on transformed cells (MICA, MICB, ULPB 1-6, CD112 and others). NK cells also mediate antibody-dependent cytotoxicity through expression of CD16 (67–70).

The limited lifespan of NK cells *in vivo* reduces their potential for long-term adverse effects such as B-cell aplasia, and NK cells are less likely to provoke GVHD than T cells in the allogeneic setting (71, 72). However, NK-mediated efficacy can be short-lived as these cells become functionally exhausted and senesce, requiring supportive cytokines like IL-2, IL-15, and/or IL-21 to survive beyond 2 weeks (73, 74). Manufacturing NK CARs can also be challenging but a recent report summarizing clinical outcomes after infusion of anti-CD19-CAR NK cells derived from umbilical cord blood demonstrated excellent results (75). In this study, anti-CD19-CAR NK cells also secreted IL-15 that prolonged persistence *in vivo*, and clinical responses were achieved with little systemic toxicity. These results will likely lead to many more NK-CAR trials in the next few years.

## Combining and Modifying Cell Administration

As an alternative approach to enhance safety and efficacy, a variety of trials combined CAR/TCR-modified cell infusion with other systemic agents or explored alternate routes of administration and infusion (**Table 5** and **Supplementary Table 4**). Drug-based strategies to mitigate severe inflammatory events like cytokine release syndrome (CRS) and associated neurotoxicity appeared initially in the setting of anti-CD19 CAR-T therapies (n= 7 including one CD19/CD20 dual receptor trial). Tocilizumab, which binds soluble and membrane-bound IL-6 receptor, was the earliest reported safety drug, first mentioned in 2016 and in 3 trials (76). Two studies have added safety management arms to their CAR studies, investigating tocilizumab prophylaxis for CRS and neurotoxic events (NCT03467256, NCT02348216) (77). Three late 2019 trials administered anakinra, an IL-1 receptor antagonist capable of crossing the blood brain barrier (78). NCT04148430 and NCT04150913 utilized prophylactic anakinra on day 0 or 2 post CAR infusion, respectively. NCT04205838 studied anakinra following onset of neurotoxicity and CRS. Registered in early 2019, one trial administered defibrotide, an oligonucleotide with protective effects on endothelial cells, from 5 days before lymphodepletion until 7 days post-CAR infusion (NCT03954106) (79).

**Table 5:** Novel combination and administration strategies in CAR and TCR therapies from 2016 to 2019.

		CAR		TCR	
*Combination with other medicines*	***SAFETY DRUGS***	Tocilizumab	n = 3		
		Anakinra	n = 3		
		Defibrotide	n = 1		
	***EFFICACY DRUGS***	Checkpoint inhibitor	n = 12	Checkpoint inhibitor	n = 9
		Interleukin-2	n = 6	Interleukin-2	n = 13
		Ibrutinib	n = 2		
		Inhibitor of Gamma Secretase	n = 1		
	***CELLS OR VIRUS***	Pulsed dendritic cells	n = 1	Pulsed dendritic cells	n = 1
		T cell antigen presenting cells expanding CAR-T	n = 1	TIL Expressing TGFbDNRII	n = 1
		Oncolytic virus	n = 1	Oncolytic virus	n = 1
*Administration*	***REGIONALLY DELIVERED***	Hepatic transarterial	n = 8		
		Intracerebral	n = 7		
		Intraperitoneal	n = 5	Intraperitoneal	n = 1
		Intra-tumoral	n = 3	Intra-tumoral	n = 1
	***INTERVENTION DESIGN***	Combined CAR injection	n = 5		
		Split dose	n = 2		
		Sequential administration	n = 1		

Drugs were also employed to mitigate negative regulation of edited cells and promote immune activity. TCR trials utilized systemic administration of checkpoint inhibitors (n=9) and IL-2 (n=13). Checkpoint inhibitors were the most studied in CAR settings and have been previously well reviewed (n=12) (47, 80). CAR trials further explored coadministration with IL-2, ibrutinib, and gamma-secretase inhibitor (n=6, 2, and 1, respectively). CAR and TCR cells were co-administered with pulsed dendritic cells, T-cell antigen presenting cells, tumor-infiltrating lymphocytes, and oncolytic viruses (n=6). Targeted regional delivery of CARs was more widely tested than with TCRs (n=22 and 2, respectively). CARs were locally supplied *via* hepatic artery (n=8), intracerebrally (n=7), intraperitoneally (n=5), and intratumorally (n=3) (**Table 5** and **Supplementary Table 4**). Of trials specifying intracerebral CAR delivery, study arms included intracavitary and/or intraventricular (n=5), intratumoral (n=1), and intracranial (n=1) injections. TCRs were administered intraperitoneally (n=1) and intratumorally in non-small cell lung cancer (n=1) (**Table 5** and **Supplementary Table 4**). CAR trials also studied alternate infusion methods for modified cells, including sequential (n=1, NCT03407859), split-dose (NCT03152435, NCT03407859), or simultaneous administration (n=8, NCT02924753, NCT03152435, NCT03407859, NCT03497819, NCT03620058, NCT03207178, NCT03549442, NCT04194931).

## Universal Allogeneic CARs

The high cost of approved autologous CAR therapies, i.e., $373,000 USD per patient for axicabtagene ciloleucel and tisagenlecleucel for adults with lymphoma and $475,000 USD for tisagenlecleucel for children and young adults with B-ALL, highlights the need for less expensive and more efficient manufacturing processes (81–83). Currently, autologous CAR cell production requires a lengthy production period ranging from 8 days when manufactured at local sites to ≥ 4 weeks when produced at commercial sites. Moreover, autologous products cannot be manufactured in time for all eligible patients, particularly individuals with rapidly progressive disease or those with insufficient peripheral immune cell numbers (84, 85). To address these issues, several groups have initiated allogeneic CAR and TCR-based trials (n=56 total), 50 of which employed CAR-T cells, 58% being anti-CD19-CARs.

Donor CARs and TCR-edited cells are promising off-the-shelf therapies if the challenges of graft versus host disease (GVHD) and short-term persistence can be mitigated by HLA-matching, specific gene knock-outs, or other cell modifications to prevent immunologic recognition and elimination of allogeneic cells in the recipient. Three allogeneic CAR trials specified HLA restrictions (NCT02050347, NCT01195480, NCT04107142). One trial initiated in 2017 described CRISPR-knockout of endogenous TCRα/β and β2-microglobulin to eliminate expression of MHC class I molecules (NCT03166878) (84). Two trials used infusion of an anti-CD52 antibody to ablate recipient lymphocytes and prevent rejection of allogeneic effector cells (NCT03939026, NCT04093596) (86). Six trials employed safety switch molecules iCasp9 (n=3, NCT01494103, NCT03056339, NCT03579927), EGFRt (n=2, NCT03114670, NCT02028455) or HSV-TK (NCT01082926) to eliminate allogeneic effector cells in the event that effector cell infusions caused severe graft versus host disease in recipients. Remaining allogeneic trials did not specify further cell modifications on *clinicaltrials.gov*.

Early results from allogeneic CAR studies are encouraging, but the number of patients treated on current trials is relatively low, and as with autologous CARs there continues to be room for further optimization. A 2019 report of anti-CD19/CD123 dual allogeneic CAR administered to three relapsed/refractory acute lymphoblastic leukemia adults achieved complete responses lasting 7–11 months (NCT03125577) (87). Cord blood-derived anti-CD19 CAR-NK cells achieved a 73% overall response in adult non-Hodgkin’s Lymphoma and chronic lymphocytic leukemia patients (NCT03056339) (75). The current status and future prospects of allogeneic CARs have been extensively reviewed (88, 89). While long term follow-up data is emerging, larger studies will be needed to establish the role of allogeneic CARs in the treatment of patients with hematologic and non-hematologic cancers.

## Non-Oncology Indications and Future Applications

Thus far, relatively few CAR and no TCR trials have focused on non-oncology indications (n=7). Four trials focused on patients with HIV, including a CAR-T construct with zinc-finger-mediated CCR5 disruption to evade HIV infection (NCT03617198). CARs were also studied in patients with Neuromyelitis Optica, Lupus Erythematosus, and Myasthenia Gravis. Lessons learned from CAR trials have already been applied to SARS-CoV-2 research. One China-based CAR trial initiated in March 2020 employs dual-targeting anti-NKG2D-ACE2 CAR-NK cells, which secretes IL-15 superagonist for NK cell longevity and has a GM-CSF neutralizing receptor to prevent cytokine release syndrome, in COVID-19 patients (NCT04324996). Based on previous success against CAR-induced cytokine storm, trials are investigating tocilizumab in the context of systemic inflammation and/or pneumonia from COVID-19 (n>10) (90, 91).

While not currently in trials at time of this review, there is considerable preclinical interest in regulatory T cell (Treg) CARs as alternatives to chronic immunosuppression in solid organ transplant and prevention and/or mitigation of autoimmune diseases like Type I Diabetes, inflammatory bowel disease, autoimmune hepatitis, encephalitis, and arthritis (92, 93). However, CAR-Treg utilization remains challenged by relatively low abundance in circulation, lack of highly efficient GMP-compliant enrichment protocols and limited stability during *in vitro* expansion (92).

## Summary

CAR and TCR therapies have birthed a promising field where initial successes for patients with unmet clinical needs have inspired further research, investment, and technological advances. The exponential growth in this field includes expansion to new and multiantigen targets, additional genetic-engineering approaches to optimize efficacy and safety, novel combination strategies, administration techniques, and exploration of different starting cell types. Late-phase studies have begun to compare anti-CD19 and anti-BCMA CAR-T therapies against current standards of care. This review of clinical trials initiated over the past 17 years demonstrates less activity in the TCR and solid tumor spaces versus CAR-T cell utilization in hematologic malignancies, though the repertoire of targeted solid tumor antigens is expanding. If CARs targeting solid tumors or for TCR-based cellular therapies can achieve similar efficacy to that seen in CARs for hematologic malignancies, dramatic increases in numbers of clinical trials and patients anticipated on these trials will certainly follow. As evident from the current distribution of early phase clinical trials, there is a great deal of international participation in the development of novel cell therapies for patients with cancer. This is truly a global effort that is supported by well-established pharmaceutical companies as well as large numbers of new biotech companies and academic investigators. China is beginning to play an important role and will continue be a major contributor to new clinical trials. This review did not cover the massive pre-clinical pipeline upstream of current clinical trials. Additionally, we excluded cell therapy modalities such as cytotoxic T lymphocytes, tumor-infiltrating lymphocytes, NK cells, immunomodulatory dendritic cell- or tumor-based vaccines. We anticipate that a similar review of commercially-approved cell therapy products and trials registered on *clinicaltrials.gov* 2 years from now will show an exponential rise in commercial cell therapy approvals and expansion of European activities in this space. Following current trends, a skew towards CARs and hematologic malignancies may continue, albeit given earlier in treatment algorithms rather than the current relapsed/refractory setting. If advances in the engineering of CARs and TCRs for solid tumors can increase the clinical efficacy of these products, clinical activity in these areas will progress rapidly and change the practice of clinical oncology forever.

## Author Contributions

LP generated, analyzed the database, and reviewed the manuscript. LP and AC generated figures and tables. AC wrote the manuscript. HT-N, SN, and JR reviewed the manuscript. All authors contributed to the article and approved the submitted version.

## Conflict of Interest

The authors declare that the research was conducted in the absence of any commercial or financial relationships that could be construed as a potential conflict of interest.
