# Supplementary material for: Global Perspective on the Development of Genetically Modified Immune Cells for Cancer Therapy
Source: Front Immunol. 2021 Feb 15;11:608485. doi: 10.3389/fimmu.2020.608485 (PMC7917113; doi:10.3389/fimmu.2020.608485)
Supplement: Supplementary file 1 [file Table_1.docx]

**Supplementary Table 1. Classification scheme for trial phases**

| Label | Sorted as |
| --- | --- |
| Early Phase 1  Phase 1  Phase 1\|Phase 2  Phase 2 | Early Phase |
| Phase 2\|Phase 3  Phase 3 | Late Phase |
| Not Applicable*  ND | Not Determined |

*“Not Applicable” Phase trials (n=29). Upon manual review of *clinicaltrials.gov* descriptions, most appear to be Phase I/II trials (i.e., primary endpoints of safety and efficacy enrolling 6 to 56 patients). “ND” Phase trials (n=2): One is CTL019 for an out-of-specification trial, the other is possibly an omission from the sponsor as this entry also did not specify patient enrollment and funding type. In this report we reclassified the only CAR trial to register as phase IV, a safety and efficacy study of an anti-CD19 CAR-T construct with only experimental arms and ten reported patients, as an early phase trial (NCT02992834).
