# Supplementary material for: Global Perspective on the Development of Genetically Modified Immune Cells for Cancer Therapy
Source: Front Immunol. 2021 Feb 15;11:608485. doi: 10.3389/fimmu.2020.608485 (PMC7917113; doi:10.3389/fimmu.2020.608485)
Supplement: Supplementary file 2 [file Table_2.docx]

**Supplementary Table 2. Classification scheme for funding source**

| Label | Sorted as |
| --- | --- |
| Other\|Industry | Industry |
| Industry | Industry |
| Industry\|Other | Industry |
| Other\|Industry\|NIH | Industry |
| Industry\|Other\|NIH | Industry |
| Other\|NIH\|Industry | Industry |
| Other | Academic |
| NIH | Academic |
| Other\|NIH | Academic |
| Other\|U.S. Fed | Academic |
| NIH\|Other | Academic |
