# Supplementary material for: Global Perspective on the Development of Genetically Modified Immune Cells for Cancer Therapy
Source: Front Immunol. 2021 Feb 15;11:608485. doi: 10.3389/fimmu.2020.608485 (PMC7917113; doi:10.3389/fimmu.2020.608485)
Supplement: Supplementary file 3 [file Table_3.docx]

**Supplementary Table 3. Novel genetic modifications in CAR and TCR therapies from 2016 to 2019**

|  | **CAR** | | **TCR** | | |
| --- | --- | --- | --- | --- | --- |
| **Resistance to negative regulation** | PD-1 knock-out | NCT03298828, NCT03747965, NCT03706326, NCT03525782, NCT04213469, NCT03545815 | PD-1 knock-out | NCT03399448 | |
|  | Anti-PD-1 expression | NCT02873390, NCT02862028, NCT03030001, NCT03615313, NCT03932955 | Anti-PD-1 expression | NCT03578406, NCT04139057 | |
|  | PD-L1 blocker expression | NCT03790891, NCT03910842,  NCT03720496, NCT03497533,  NCT04191941 |  | | |
|  | Anti-PD-1 and anti-CTLA-4 expression | NCT03182816, NCT03179007,  NCT03182803 |  |  |  |
|  | PD-1 Fc fusion protein secreted | NCT04163302, NCT04162119 |  |  |  |
|  | PD-1 shRNA expression | NCT03208556 |  |  |  |
|  | Activated cytoplasmic PD-1 | NCT03540303 |  |  |  |
|  | TGF-beta resistance | NCT03089203 |  |  |  |
|  | Endogenous HPK1 disruption | NCT04037566 |  |  |  |
| **Interleukin expression** | IL15 | NCT03579927, NCT03721068, NCT03579888, NCT03774654, NCT03294954 |  |  |  |
|  | IL-7 and CCL19 | NCT03929107, NCT03778346 |  |  |  |
|  | IL-7 receptor | NCT03635632, NCT04099797 |  |  |  |
|  | IL-15 or both IL-15 and IL-21 | NCT04093648 |  |  |  |
|  | IL-12 | NCT03542799 |  |  |  |
|  | IL-7 and CCL19 or IL12 | NCT03932565 |  |  |  |
| **Tumor homing** | CCR4 expression | NCT03602157 |  |  |  |
|  | CXCR5 modified | NCT04153799 |  |  |  |
| **Fratricide resistance** | CD7 knock-out CD7-targeting CAR | NCT03690011 |  |  |  |
| **Safety switch** | iCasp9 | NCT03330691, NCT03103971, NCT03579888, NCT03710421, NCT04185038, NCT03070327, NCT04109482, NCT03085173, NCT03244306, NCT02937844, NCT02706405, NCT03114670, NCT03084380 | iCasp9 | NCT02743611 | |
|  | EGFRt | NCT03103971, NCT03710421, NCT04185038, NCT03070327, NCT04109482, NCT03085173, NCT03244306, NCT02937844, NCT02706405, NCT03114670, NCT03084380, NCT03263208, NCT03618381 |  | | |
|  | EGFRt and HERt on two different CARs | NCT03330691 |  |  |  |
|  | Herpes simplex virus thymidine kinase | NCT04097301 |  |  |  |
|  | RQR8 | NCT03590574 |  |  |  |
| **Virus resistance** | Resistant to HIV by CCR5 modification | NCT03617198 |  |  |  |
| **Immune resistance** | β2m and TCR disruption | NCT03166878 | TCRα/β disruption | | NCT03399448 |
