# Supplementary material for: Global Perspective on the Development of Genetically Modified Immune Cells for Cancer Therapy
Source: Front Immunol. 2021 Feb 15;11:608485. doi: 10.3389/fimmu.2020.608485 (PMC7917113; doi:10.3389/fimmu.2020.608485)
Supplement: Supplementary file 4 [file Table_4.docx]

**Supplementary Table 4. Novel combination and administration strategies in CAR and TCR therapies from 2016 to 2019**

|  | | **CAR** | | | **TCR** | |
| --- | --- | --- | --- | --- | --- | --- |
| *Combination with other medicines* | **Safety drugs** | Tocilizumab | | NCT03467256, NCT04007029, NCT02906371 |  | |
|  |  | Anakinra | | NCT04205838, NCT04148430, NCT04150913 |  |  |
|  |  | Defibrotide | | NCT03954106 |  |  |
|  | **Efficacy drugs** | Checkpoint inhibitor | | NCT02926833, NCT04003649, NCT04181827, NCT03287817, NCT03726515, NCT03630159, NCT04134325, NCT02706405, NCT03310619, NCT03704298, NCT03525782, NCT03628053 | Checkpoint inhibitor | NCT03168438, NCT03697824  NCT03406858, NCT03709706  NCT03070392, NCT02775292  NCT04146298, NCT03907852  NCT03747484 |
|  |  | Interleukin-2 | | NCT02850536, NCT02830724, NCT03415100, NCT03098355, NCT03818165, NCT04119024 | Interleukin-2 | NCT03017131, NCT03197025  NCT03240861, NCT03190941  NCT03450122, NCT03412877  NCT02858310, NCT02770820  NCT02774291, NCT03691376  NCT03506802, NCT03745326  NCT03891706 |
|  |  | Ibrutinib | | NCT03331198, NCT03960840 |  | |
|  |  | Inhibitor of Gamma Secretase | | NCT03502577 |  |  |
|  | **Cells or virus** | Pulsed dendritic cells | | NCT03291444 | Pulsed dendritic cells | NCT02775292 |
|  |  | T cell antigen presenting cells expanding CAR-T | | NCT03186118 | TIL Expressing TGFbDNRII | NCT02650986 |
|  |  | Oncolytic virus | | NCT03740256 | Oncolytic virus | NCT00704938 |
| *Administration* | **Regionally delivered** | Hepatic transarterial | | NCT03993743, NCT03818165, NCT04037241, NCT02850536, NCT03370198, NCT03349255, NCT02715362, NCT02706782 |  | |
|  |  | Intracerebral | Intracavitary, intraventricular | NCT04185038, NCT03638167, NCT03500991 NCT04077866­­, NCT03696030 |  |  |
|  |  |  | Intratumoral | NCT03283631 |  |  |
|  |  |  | Intracranial | NCT04003649 |  |  |
|  |  | Intraperitoneal | | NCT03682744, NCT03585764, NCT03563326, NCT03608618, NCT03907527 | Intraperitoneal | NCT03017131 |
|  |  | Intra-tumoral | | NCT02862704, NCT03130712, NCT03184753 | Intra-tumoral | NCT03778814 |
|  |  | Combined CAR injection | | NCT03497819, NCT03620058  NCT03207178, NCT03549442  NCT04194931 |  | |
|  | **Intervention design** | Split dose | | NCT02924753  NCT03152435 |  |  |
|  |  | Sequential administration | | NCT03407859 |  |  |
